# Epitopes of anti-RIFIN antibodies and characterization of *rif*-expressing *Plasmodium falciparum* parasites by RNA sequencing

**DOI:** 10.1038/srep43190

**Published:** 2017-02-24

**Authors:** Jun-Hong Ch’ng, Madle Sirel, Arash Zandian, Maria del Pilar Quintana, Sherwin Chun Leung Chan, Kirsten Moll, Asa Tellgren-Roth, IngMarie Nilsson, Peter Nilsson, Ulrika Qundos, Mats Wahlgren

**Affiliations:** 1 Department of Microbiology, Tumor and Cell Biology (MTC), Karolinska Institutet, Stockholm, Sweden; 2 Department of Microbiology and Immunology, National University of Singapore, Singapore; 3 Affinity Proteomics, Science for Life Laboratory, School of Biotechnology, Royal Institute of Technology (KTH), Stockholm, Sweden; 4 Center for Biomembrane Research, Department of Biochemistry and Biophysics, Stockholm University, Stockholm, Sweden

## Abstract

Variable surface antigens of *Plasmodium falciparum* have been a major research focus since they facilitate parasite sequestration and give rise to deadly malaria complications. Coupled with its potential use as a vaccine candidate, the recent suggestion that the repetitive interspersed families of polypeptides (RIFINs) mediate blood group A rosetting and influence blood group distribution has raised the research profile of these adhesins. Nevertheless, detailed investigations into the functions of this highly diverse multigene family remain hampered by the limited number of validated reagents. In this study, we assess the specificities of three promising polyclonal anti-RIFIN antibodies that were IgG-purified from sera of immunized animals. Their epitope regions were mapped using a 175,000-peptide microarray holding overlapping peptides of the *P. falciparum* variable surface antigens. Through immunoblotting and immunofluorescence imaging, we show that different antibodies give varying results in different applications/assays. Finally, we authenticate the antibody-based detection of RIFINs in two previously uncharacterized non-rosetting parasite lines by identifying the dominant *rif* transcripts using RNA sequencing.

The function of repetitive interspersed families of polypeptides (RIFINs) in blood group A-specific rosetting has generated much interest concerning the importance of malaria in shaping geographical blood group profiles^1^. However, the number (150–200 copies per haploid genome) and diversity of this multigene copy family suggests that the characteristics of these proteins are likely to be highly varied^2^,^3^,^4^,^5^.

RIFINs have been classified into two subgroups, with 70% belonging to subgroup A (A-RIFINs), possessing a 25 amino acid insertion-deletion (indel) region, and 30% to subgroup B (B-RIFINs) which lack an indel^3^. During the trophozoite stages, A-RIFINs have been shown to be exported to the host cell membrane while B-RIFINs remain within the parasite^3^,^6^,^7^,^8^. RIFINs have also been shown to be expressed in sporozoite, merozoite and gametocyte stages although their functions there have yet to be elucidated^8^,^9^,^10^.

Rosettes are formed when an infected erythrocyte (iRBC) binds to uninfected erythrocytes (uRBCs) to form cell clusters which then occlude microvasculature and lead to malaria complications. The rosettes formed by blood group A erythrocytes are of clinical significance because individuals with blood group A are more likely to develop severe malaria compared to blood group O^11^,^12^,^13^,^14^,^15^,^16^,^17^. Rosettes of this blood group have been demonstrated to have stronger binding^18^, be resistant to heparin-induced dispersion^19^ and even to shield the iRBC from antibody binding^20^.

Another report on broadly reactive human anti-RIFIN antibodies has also generated much discussion about the potential for RIFINs as vaccine candidates^21^. Spontaneously occurring LAIR1 insertions between V and DJ segments gave rise to a 98 amino acid collagen-binding domain insertion that resulted in broadly neutralizing antibodies directed towards the RIFINs on the iRBC surface.

With the great promise for medical advances captured by these early findings, it is fundamental to assess the quality of available reagents to assay for RIFINs. Thus far, most studies on RIFINs make use of techniques like surface iodination or antibody-based assays (western blotting, immunofluorescence microscopy, flow cytometry, immunoprecipitation etc.) to study the presence, localization or function of RIFINs in the parasite^1^,^7^,^10^,^21^,^22^,^23^,^24^,^25^,^26^,^27^. The specificities of such approaches are limited and are only occasionally backed with more unambiguous methods like GFP-tagged over-expression models or MALDI-TOF identification of immuno-precipitated proteins. To the best of our knowledge, anti-RIFIN antibody profiling is never performed though these reagents are linchpins to study claims. As described recently^28^,^29^, the lack of thorough antibody validation will inadvertently lead to experimental results that are irreproducible, confusing or untrue.

In this study, we make use of ultra-dense peptide arrays to examine the specificities of anti-RIFIN IgG preparations, test the functionality of these antibodies in different antibody-based assays, and finally follow up with RNA sequencing (RNAseq) to determine RIFIN expression in laboratory-adapted parasite lines. In conclusion, we describe the assay-specific utility of the antibodies, the potential for cross-reactivity, and also identify the dominant RIFINs on two non-rosetting parasite lines.

## Results

### Western blots

**Figure 1:**
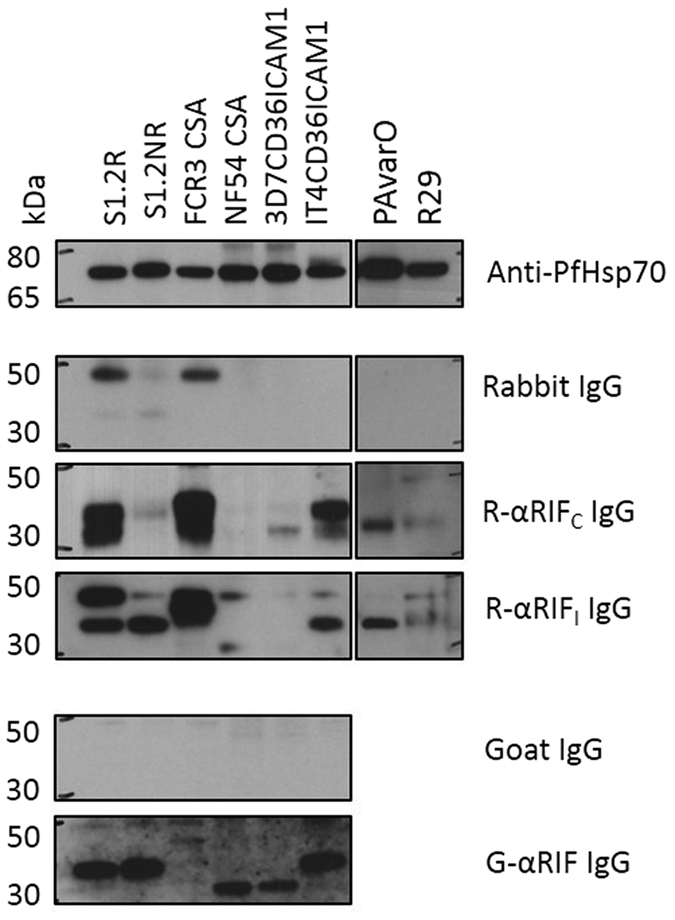
Western blots of RIFINs in multiple parasite strains. SDS-extracted parasite lysates of S1.2R, S1.2NR, FCR3CSA, NF54CSA, 3D7CD36ICAM1 and IT4CD36ICAM1 were run on SDS-PAGE (lanes 1-6 respectively), transferred to nitrocellulose membrane and blotted with rabbit anti-PfHsp70 (1:2000), non-immune rabbit IgG (10 μg/ml), RαRIF_C_ (rabbit anti-A-RIFIN C-terminus) IgG (10 μg/ml), RαRIF_I_ (rabbit anti-A-RIFIN indel) IgG (10 μg/ml), non-immune goat IgG (10 μg/ml) and GαRIF (goat anti-A-RIFIN full length) IgG (10 μg/ml). Corresponding HRP-conjugated secondary anti-rabbit and anti-goat IgG antibodies were used together with ECL reagent for detection. Description of the antigens used for immunizations of RαRIF_C_, RαRIF_I_ and GαRIF are described in Supplementary Table S1 and diagrammatically illustrated in Fig. 3D.

Several techniques were used to characterize anti-RIFIN antibodies. To begin, purified IgG from 10 rabbits (RαRIF_C_, R2αRIF_C_, R3αRIF_C_, R4αRIF_C_, R5αRIF_C_, R6αRIF_C_, R7αRIF_C_, R8αRIF_C_, RαRIF_I_ and R2αRIF_I_) and 1 goat (GαRIF) that had been immunized with RIFIN peptides/protein (Supplementary Table S1) were tested in Western blots of SDS extracts of S1.2 R, a well-studied rosetting parasites strain that is RIFIN-positive (results not shown). Only antibodies from RαRIF_C_, RαRIF_I_ and GαRIF resulted in bands of the expected size of approximately 35 kDa (Fig. 1). Together with the relevant commercial non-immune IgG to exclude non-specific staining and anti-Hsp70 to ensure similar loading (Fig. 1), these three reactive IgG preparations were used to detect the presence of RIFINs in other laboratory strains including FCR3CSA, NF54CSA, 3D7CD36ICAM1, IT4CD36ICAM1, PAvarO and R29.

Western blot staining with RαRIF_C_ IgG (rabbit immunized with the conserved A-RIFIN C-terminal peptide, see Fig. 3D) showed prominent bands with sizes corresponding to the RIFINs (between 30–40 kDa) in S1.2 R, FCR3CSA, IT4CD36ICAM1 and to a lesser extent in PAvarO (Fig. 1 and Supplementary Figure S1). In these lysates, the presence of two bands between 35–45 kDa was noted for S1.2 R and FCR3CSA. Several faint bands at higher molecular weights could also be observed and were likely due to some limited degree of cross-reactivity (Supplementary Figure S1).

Staining with RαRIF_I_ IgG (rabbit immunized with a conserved A-RIFIN indel peptide, see Fig. 3D) showed that A-RIFINs were expressed in S1.2 R, FCR3CSA, IT4CD36ICAM1 and PAvarO, but also to some degree in S1.2NR and R29 (Fig. 1 and Supplementary Figure S1). Again, some faint bands could again be seen (in S1.2 R > 185 kDa and in FCR3CSA at 115 kDa and >185 kDa) and may indicate some cross reactivity to higher molecular weight proteins (Supplementary Figure S1).

IgG purified from GαRIF (goat immunized with full-length PF3D7_0100400, see Fig. 3D) showed prominent bands at about 35 kDa for S1.2 R, S1.2NR and IT4CD36ICAM1 (Fig. 1). While there was only a single clear band for S1.2 R, there were multiple prominent bands at 30, 50, 60, 80, 115 and 185 kDa in extracts of other parasite lines (Supplementary Figure S1) indicating significant cross-reactivity with other parasite proteins in these parasites.

### Indirect Immunofluorescence Assay

**Figure 2:**
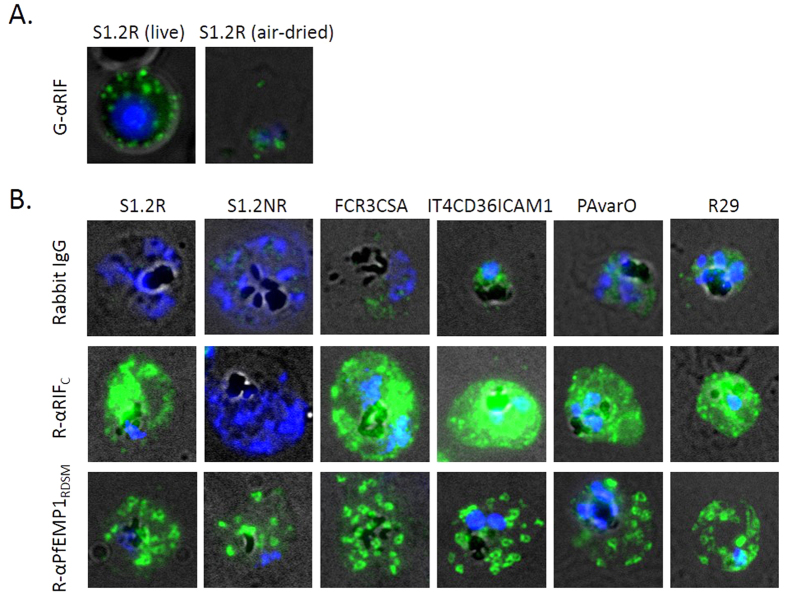
Indirect immunofluorescence micrographs. (**A**) GαRIF IgG labelling of RIFINs on a live S1.2R-infected erythrocyte and an air-dried iRBC. (**B**) Air-dried monolayers of erythrocytes infected with S1.2R, S1.2NR, FCR3CSA, IT4CD36ICAM1, PAvarO and R29 parasites stained with non-immune rabbit IgG (10 μg/ml), anti-RIFIN RαRIF_C_ rabbit IgG (10 μg/ml) and Maurer’s cleft labelling by R-αPfEMP1_RDSM_ IgG (8 μg/ml). Alexa488-conjugated secondary anti-rabbit or anti-goat antibodies were used (1:200) as well as Vectashield with DAPI (nuclear staining). Merged channels of transmission light (black and white), green (Alexa 488) and blue (DAPI) and representative cells are shown here.

GαRIF IgG had previously been used to label live S1.2 R parasites for cytometry^1^ and its reactivity has been very specific – no other laboratory strain or field isolate tested to date have shown any surface staining (results not shown). Unlike the antibody labeling of live S1.2R-infected cells which showed punctate surface-specific staining (Fig. 2A, left panel), air dried monolayers stained with GαRIF IgG showed only a faint parasite cytoplasmic staining (Fig. 2A, right panel) suggesting that the native conformational (3D) epitope may have been lost during the fixing by desiccation. Higher antibody concentrations resulted in non-specific staining of even the uninfected RBCs (data not shown) and did not improve detection.

Since desiccation results in breaks in the membrane and exposes the inner surface of the RBC membrane where the conserved RIFIN C-terminus is located, RαRIF_C_ IgG could readily label S1.2R-infected RBCs in a patchy manner (Fig. 2B). In comparison, IgG from RαRIF_I_, R5αRIF_C_ and R6αRIF_C_ showed no notable staining (data not shown). RαRIF_C_ IgG was then applied to air-dried monolayers of other parasite strains to detect and localize potential A-RIFINs. While S1.2NR (Fig. 2B), NF54CSA and 3D7CD36ICAM1 (data not shown) had no notable staining, FCR3CSA, IT4CD36ICAM1, PAvarO and R29 parasites showed staining similar to S1.2 R (Fig. 2B).

The staining by RαRIF_C_ IgG was markedly different from that of R-αPfEMP1_RDSM_ IgG, which stained all the six tested parasites with distinct “donut” shapes (Fig. 2B). This strain-transcending rabbit αPfEMP1_RDSM_ (respiratory distress severe malaria) antibody has been described before as staining the Maurer’s clefts though binding the RDSM peptide of PfEMP1 that is only exposed *en route* to the RBC surface^30^. In comparison, non-immune rabbit IgG at the same concentration showed little or no staining of the air-dried monolayers (Fig. 2B).

### Epitope region mapping by peptide array

An ultra-dense peptide array was used to epitope map anti-RIFIN polyclonal IgG antibodies purified from the sera of two non-immune and five immunized animals (RαRIF_C_, R5αRIF_C_, R6αRIF_C_, RαRIF_I_, and GαRIF). The peptide array contained 12 amino acid fragments of parasite surface antigens including PfMC-2TM, PHIST, RIFIN, STEVOR, SURFIN and several PfEMP1 proteins, with an 11-residue overlap between peptides derived from the same protein.

Background binding was minimal: the non-immune rabbit IgG reacted with 7 of the 443 arrayed proteins (reactivity scores of 2 or 3) and non-immune goat IgG reacted with only one protein (reactivity score of 2).

**Figure 3:**
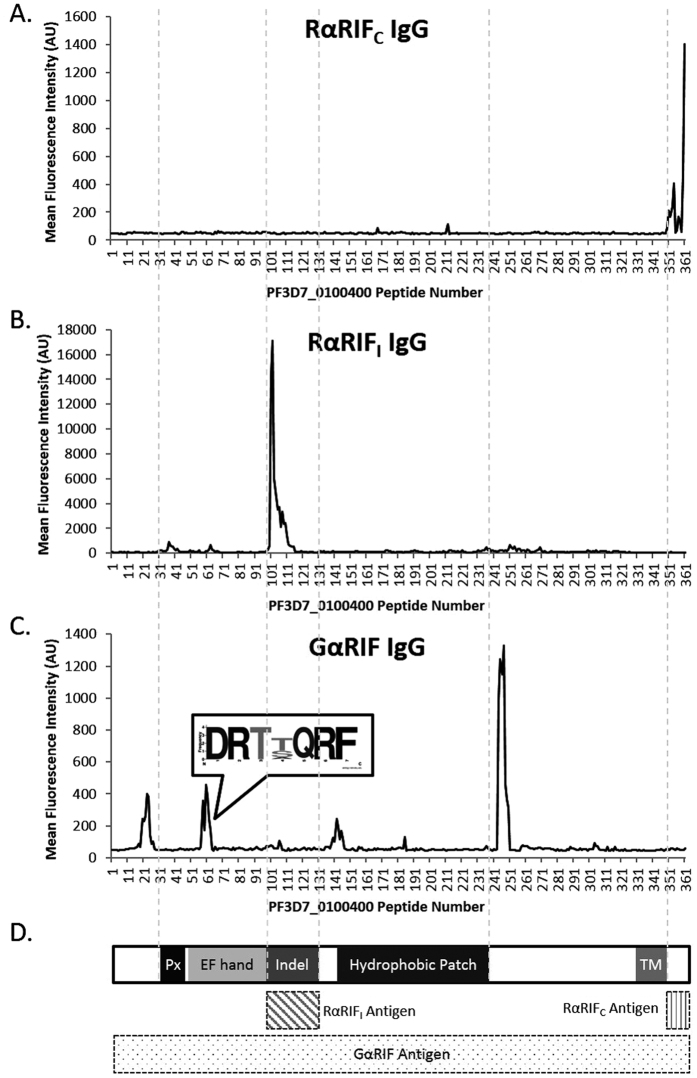
Epitope region mapping of anti-RIFIN antibodies using an ultra-dense peptide array. The reactivity for polyclonal IgG purified from (**A**) RαRIF_C_, (**B**) RαRIF_I_ and (**C**) GαRIF are shown here against peptides from the RIFIN protein PF3D7_0100400. Y-axis shows mean median fluorescence intensity of duplicate spots and X-axis shows the peptide number from the N terminus to the C-terminus. (**C**) A conserved amino acid sequence that is recognized by GαRIF IgG is highlighted. (**D**) A diagram of RIFIN PF3D7_0100400 domains is provided together with the respective antigens used for immunizations: RαRIF_C_ was immunized with the C-terminus, RαRIF_I_ with the indel region and GαRIF with the full length of PF3D7_0100400. The abbreviation “Px” stands for PEXEL motif while “TM” refers to the transmembrane domain. Detailed intensity plots of the key epitope regions are highlighted in Supplementary Figure S2.

The IgG of RαRIF_C_, immunized with the conserved 20 amino acid C-terminal peptide of A-RIFINs, showed that about half of the RIFINs (141/278) were bound at the C-terminus after the transmembrane (TM) segment with a most frequent reactivity score of 6 (Supplementary Data S1). Of these 141 RIFINs, RαRIF_C_ gave a reactivity score of 8 for PF3D7_0100400, with the 8 peptides spread across three epitope regions (Fig. 3A and Supplementary Figure S2A). Several SURFINs and STEVORs also showed reactivity, but RαRIF_C_ had no reactivity against any of the arrayed PfEMP1s, PHISTs or PfMC2TMs.

Both R5αRIF_C_ and R6αRIF_C_ were immunized with the conserved B-RIFIN C-terminus. However, IgG of both rabbits had a higher reactivity score for many of the short-listed PfEMP1s than the RIFINs (Supplementary Data S1). There was also significant cross-interaction with SURFIN and PHIST peptides which limited the utility of these unspecific antibodies.

RαRIF_I_, immunized with the semi-conserved indel region of A-RIFINs, bound a third of the RIFINs (102/278) with reactivity scores around 3 to 11 (Supplementary Data S1). Most of these RIFINs that reacted were a subset of those detected by RαRIF_C_ IgG and binding was generally localized to the indel region. In PF3D7_0100400, RαRIF_I_ recognized 13 peptides that were confined within one epitope region (Fig. 3B and Supplementary Figure S2B). There was a limited degree of cross-reactivity to a few other members from surface antigen families.

GαRIF was immunized with full length A-RIFIN PF3D7_0100400 and had a reactivity score of 23 against the peptides of this same protein (Supplementary Data S1). Closer inspection of the peptide-intensity plot of this 372 amino acid sequence revealed five main epitopes regions (Fig. 3C and Supplementary Figure S2C): a sequence upstream of the PEXEL motif (NTHKKPSITSRHIQTTR –unique to PF3D7_0100400), a sequence in the first half of the EF hand region (MQQFHDRTTQRFHEYDE), two adjoining sequences at the start of the hydrophobic patch (QNLGKIVAPSSGVLAG –unique to PF3D7_0100400) and a final region just after the hydrophobic patch (PSLVNDQLVGTFNTSDPF –unique to PF3D7_0100400). Of the 436 bona fide peptides GαRIF reacted towards, 336 peptides coming from 52 RIFINs contained the sequence D-R-T-T/S/A-Q-R-F (Fig. 3C insert and Supplementary Data S1). In total, GαRIF IgG reacted with a quarter of all the RIFINs on the array (66/278) of which most had a reactivity score of 6. Binding to a few members of PfEMP1, SURFINs, STEVORs and PHISTs could also be detected.

### RNA sequencing and qPCR validation

To characterize RIFIN expression, RNAseq was performed on NF54CSA, S1.2NR, FCR3CSA and IT4CD36ICAM1 parasites at 10, 20, 30 and 40 hours post invasion (hpi), with the exception of S1.2NR at 40 hpi due to failed library preparation. RNAseq was not carried out for PAvarO as its genome is not well curated and not for 3D7CD36ICAM1 or R29 as they did not have high levels of RIFINs as indicated by Western blot.

**Figure 4:**
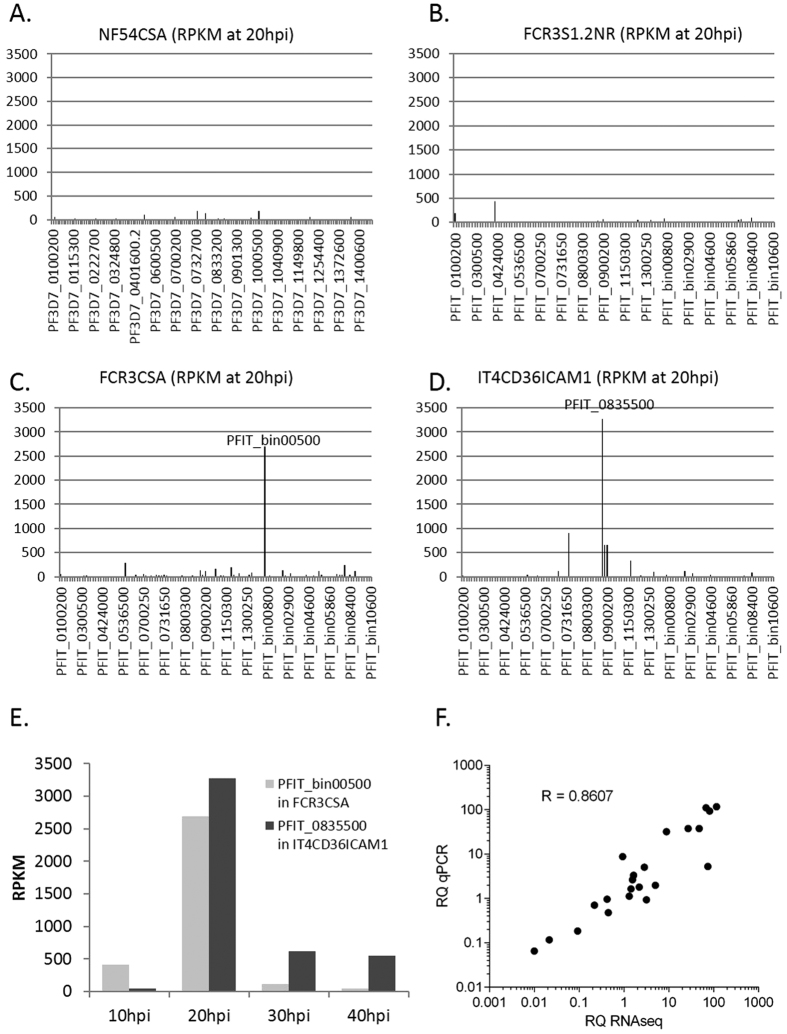
RNA sequencing counts of *rif* genes. RNA from (**A**) NF54CSA, (**B**) S1.2NR, (**C**) FCR3CSA and (**D**) IT4CD36ICAM1 were extracted at four time points and sent for RNA sequencing. Results for the *rif* genes are presented here in RPKM at 20 hpi. (**E**) RPKM values at 10, 20, 30 and 40 hpi are provided to show the temporal expression of the two dominant *rif* genes (PFIT_bin00500 in FCR3CSA and PFIT_0835500 in IT4CD36ICAM1). (**F**) Quantitative real time PCR was performed to validate the RNA sequencing results and Pearson’s R shows level of correlation between the relative quantity (RQ) of qPCR fold expression and RNAseq RPKM values.

In NF54CSA and S1.2NR parasites (both negative for RIFINs using RαRIF_C_ antibodies) the Reads Per Kilobase of transcript per Million mapped reads (RPKM) of *rif* genes were relatively low (Supplementary Data S2). In NF54CSA, the highest expressed *rifs* were PF3D7_120050 (239 RPKM at 10 hpi, Supplementary Data S2), PF3D7_1000600 (188 RPKM at 20hpi), PF3D7_0732900 (183 RPKM at 20 hpi), PF3D7_0808900 (129 RPKM at 20 hpi) and PF3D7_0425900 (103 RPKM at 20 hpi) (Fig. 4A, Supplementary Data S2). In S1.2NR, the highest expressed *rifs* were PFIT_0411800 (431 RPKM at 20 hpi) and PFIT_0100200 (190 RPKM at 20 hpi) (Fig. 4B, Supplementary Data S2).

In contrast, the RPKM of the highest expressed *rifs* in FCR3CSA and IT4CD36ICAM1 (both positive for RIFINs using RαRIF_C_ antibodies) were markedly higher (Supplementary Data S2). The highest expressed *rif* for FCR3CSA was PFIT_bin00500 (2693 RPKM at 20 hpi) followed by PFIT_0536600 (281 RPKM at 20 hpi) and PFIT_bin07200 (243 RPKM at 20 hpi) (Fig. 4C). At similar levels, the highest expressed *rif* in IT4CD36ICAM1 was PFIT_0835500 (3273 RPKM at 20 hpi) followed by PFIT_0733200 (910 RPKM at 20 hpi), PFIT_0900200 (662 RPKM at 20 hpi) and PFIT_0900150 (660 RPKM at 20 hpi) (Fig. 4D). Temporally, the peak expression for these two maximally expressed RIFINs in these two parasites was at 20 hpi (Fig. 4E).

To validate the RNAseq results, ten *rif* genes were selected for verification by qPCR using fructose bisphosphate aldolase (FBA) as a reference (Fig. 4F). There was a strong correlation (Pearson r = 0.8607) between RNAseq and qPCR data from the three tested parasite lines (S1.2NR, FCR3CSA and IT4CD36ICAM1) thereby validating RNAseq quality and confirming that the dominant *rif* transcripts are PFIT_bin00500 and PFIT_0835500 for FCR3CSA and IT4CD36ICAM1 respectively.

## Discussion

Despite being the largest multigene family of proteins in *P. falciparum*, the RIFINs have remained poorly characterized and have only come into the research spotlight most recently. Among other factors, the sheer number of genes and the lack of reliable reagents to assay these proteins have hampered advances in this field of research.

Previous attempts to generate high quality antibodies had limited success. For example, we had tried to generate mice hybridomas (to get monoclonal antibodies) by first immunizing 6 mice with the near-full length PF3D7_0100400 protein (amino acid 30-329), 6 mice with a semi-conserved A-RIFIN N-terminal peptide (PSNYDNDPEMKEVMQ), 3 mice with a conserved B-RIFIN N-terminal peptide (FDRQTSQRFEEYEER) and 3 mice with A-RIFIN C-terminus (LRYRRKKKMKKKLQYIKLL). Although positive to the antigens by ELISA, none of the resulting antisera were reactive against S1.2 R parasites by live cell surface staining (native extracellular epitopes), IFA (fixed extracellular and intracellular epitopes) or Western blot (linearized epitopes) (results not shown).

Another series of immunizations in ten rabbits and one goat yielded some RIFIN-reactive antisera, some of which were published previously^1^. In the present study we apply these reagents to multiple laboratory parasite strains and map the epitopes of these anti-RIFIN antibodies.

Of the polyclonal IgG purified from sera of these eleven animals, only three (RαRIF_C_, RαRIF_I_ and GαRIF) gave western blot bands corresponding to the expected size of the dominant RIFIN in FCR3S1.2 (PFIT_bin05750 predicted to be 37.1 kDa). These antibodies were then tested on the SDS extracted lysates of seven other strains (S1.2NR, NF54CSA, FCR3CSA, 3D7CD36ICAM1, IT4CD36ICAM1, PAvarO and R29) but with inconsistent results. Some lysates had bands from all three antibodies; others had no bands, while some showed bands only for one or two of the antibodies. The inconsistencies are perhaps unsurprising since the polyclonal antibodies were raised against different epitopes. Even if RIFINs are being expressed, they may or may not have the same epitopes and as such may not be recognized by the antibodies. Also, the appearance of multiple close bands had also been described before^1^ but the reason for this has yet to be uncovered. Since the specificity of the antibodies had yet to be ascertained at this stage, we could only conclude tentatively that S1.2 R, S1.2NR, FCR3CSA, IT4CD36ICAM1, PAvarO and R29 might contain RIFINs while NF54CSA and 3D7CD36ICAM1 had no detectable RIFINs.

In immunofluorescence staining, only one of these three antibodies (RαRIF_C_) gave a positive signal to S1.2 R parasites. When applied to the same seven parasite strains as before, iRBC membrane staining could be identified in FCR3CSA, IT4CD36ICAM1, PAvarO and R29 but not in S1.2NR, NF54CSA or 3D7CD36ICAM1. The pattern of localization of all five positively-stained parasites closely resembled each other with patchy staining of the iRBC surface. The staining was markedly different from the characteristic donut-shaped pattern observed by staining of the Maurer’s clefts^30^ and appears contrary to previous findings that indicate RIFIN export via the Maurer’s clefts^24^,^25^,^27^. The difference in staining may be because of sub-detectable levels of RIFINs located in the Maurer’s clefts at the point of staining, because the compartmentalization of RIFINs within the Maurer’s clefts are unique in different parasites, or possibly because the RIFINs of these parasites are trafficked to the RBC surface without passing through the Maurer’s clefts. However, it is difficult to draw conclusions since co-staining was not performed (both R-αPfEMP1_RDSM_ and RαRIF_C_ are rabbit IgG making co-staining challenging) and once again, the specificity of RαRIF_C_ IgG could be called into question. As such, peptide arrays were utilized to determine the specificity of the three most promising antibodies and provide information on their targeted epitopes.

An ultra-dense peptide array was designed to contain peptides spanning most of the known *P. falciparum* surface antigens. Of the anti-RIFIN antibodies tested, RαRIF_C_ was the most specific for the RIFINs, binding to the conserved C-terminus of half of the tested RIFINs and not to many other peptides on the array. This suggests that RαRIF_C_ antibodies have high specificity for RIFINs since cross-reactivity to other tested surface antigens is nominal, at least at the level of the primary amino acid sequence. Western blots of the parasite SDS extract also had no prominent bands outside of the 35–45 kDa region, further supporting the specificity of RαRIF_C_ IgG to the RIFINs. Importantly, the peptide array indicated that RαRIF_C_ IgG should have a 50% likelihood of detecting a randomly expressed RIFIN - a considerable percentage given the variability of the RIFINs. Implicitly, it also does mean that there is a 50% chance that a RIFIN-expressing parasite would be missed as a false negative. However, RαRIF_C_ antibodies are not useful for functional assays (they cannot be used to block the function of the protein in inhibition assays) since the binding is specific to the intracellular C-terminus and there is therefore a requirement for cells to first be fixed and permeabilized.

RαRIF_I_ antibodies also demonstrated good specificity for RIFINs based on the peptide array, with binding primarily targeting the indel regions of a third of all RIFINs arrayed. Although this region is predicted to be extracellular, RαRIF_I_ showed no binding to live iRBC surface of S1.2, FCR3CSA, IT4CD36ICAM1, PAvarO or R29. Since RαRIF_I_ was able to give prominent bands to lysates of those same parasites by Western blot, this may suggest that the epitope is hidden in the native protein conformation and only accessible to antibody binding when linearized. This thereby renders RαRIF_I_ antibodies unsuitable for functional studies as well.

The only functional anti-RIFIN antibody at our disposal is from GαRIF – it is able to bind to the live S1.2 R infected erythrocyte surface and disrupt blood group A rosettes^1^. GαRIF antibodies show only one band in Western blots of S1.2 R lysate and are highly specific to S1.2 R when used in live cell surface staining; no labeling of any other tested parasite lines has been observed to date. Based on the peptide array, the reactivity of GαRIF antibodies was also primarily towards the RIFINs but with some cross-reactivity to other surface antigens. This could be because a much longer full length RIFIN was used in the immunization (as opposed to a peptide antigen in the case of RαRIF_C_ and RαRIF_I_) and may have consequently yielded a greater diversity of antibodies with increased cross-reactivity. This would also explain why multiple bands feature prominently on several parasites’ SDS extracts that are beyond the expected sizes of the RIFINs. Notwithstanding, there were five main epitopes spread across the protein length, of which three were unique to the RIFIN used in the immunization (PF3D7_0100400). Only one epitope with the sequence DRT(T/S/A)QRF (located extracellularly in the first half of the EF-hand domain) reacted against a quarter of all the RIFINs arrayed.

Having validated the specificities of the antibodies and shortlisted four additional parasite strains that were likely to be RIFIN-positive (positive by Western and IFA using RαRIF_C_), we proceeded with RNA sequencing of two of these strains with the IT4 background to identify which one/ones of the 150-200 *rifs* was expressed. As a surface exposed antigen susceptible to immune recognition, the *rifs* are believed to be expressed in an allelic exclusive manner. This has been demonstrated in the S1.2 R transcriptome which showed a single dominantly-expressed *rif* (PFIT_bin05750)^1^. In this study, we observe that two additional RIFIN-positive parasite lines also transcribe a single dominant *rif*. In FCR3CSA, the *rif* transcript with the highest RPKM value was PFIT_bin00500, with a value of 2693 that was almost 10-fold higher than the next highest (PFIT_0536600 with RPKM of 281). Similarly, another single dominant *rif* was found in IT4CD36ICAM1, with PFIT_0835500 having a RPKM value of 3273 that was almost four-fold higher than the next highest *rif* transcript. The peak expression of these dominant *rif* transcripts all occurred at 20 hpi, suggesting that their protein products may be relevant in the mature asexual parasite stages. In comparison, the two parasite strains that were likely to be RIFIN-negative (negative by Western and IFA using RαRIF_C_) were also sent for RNA sequencing and both showed much lower RPKM values for all *rifs* at 20 hpi (all *rif* RPKM below 500).

Like the dominant *rif* of S1.2 R, the dominant *rif* of FCR3CSA and IT4CD36ICAM1 both have C-terminal sequences containing the motif KKKLQYIK (Supplementary Table S2). This motif was also present on the antigen used for immunizing RαRIF_C_ and shown to be an epitope region by the ultra-dense peptide array. This therefore explains the reactivity of RαRIF_C_ IgG to the dominant RIFINs of S1.2 R, FCR3CSA and IT4CD36ICAM1 by Western blot and IFA. Although the same motif is present on the ‘dominant’ (highest RPKM at 20 hpi) *rif* of S1.2NR, it is likely that the relatively low RPKM value (13% and 16% of the RPKM of dominant *rif* of FCR3CSA and IT4CD36ICAM1 respectively) and correspondingly lower protein levels resulted in a lack of detection. The ‘dominant’ *rif* of NF54CSA does not have this motif and was not expected to be reactive.

The sequences of the dominant RIFINs of FCR3CSA and IT4CD36ICAM1 were aligned with the two previously studied RIFINs (PFIT_bin05750 and PF3D7_0100400) using Clustal Omega (Supplementary Figure S3). Using the predictor of apparent free energy difference, ΔG_app_ (http://dgpred.cbr.su.se)^31^ PFIT_bin00500 and PFIT_0835500 were predicted to contain 3 possible transmembrane regions: the signal peptide, the hydrophobic patch and transmembrane (TM) domain.

Located upstream from the PEXEL motif, the signal peptides of PFIT_0835500 and PFIT_bin00500 have ΔG_app_ value of 2.71 and 2.87 respectively. This is slightly higher than the predicted ΔG_app_ values of the signal peptide of 3D7_0100400 and PFIT_bin05750 (2.22 and 2.53 respectively). The latter two have been shown experimentally to integrate into the ER membrane to at least 70% in an N_cyt_-C_lum_ orientation (manuscript in preparation) and it is possible that the signal peptide of PFIT_0835500 and PFIT_bin00500 will be inserted likewise. Nevertheless, such signal sequences in *Plasmodium* have proved difficult to predict reliably^32^.

Both PFIT_bin00500 and PFIT_0835500 have predicted hydrophobic patches. Though these moderately hydrophobic regions that can be forced into the ER membrane by neighboring TM regions^33^, our recent findings suggest that they are more likely to act as a reentrant loop or could interact with other molecules (manuscript in preparation).

To our knowledge, all RIFINs have a highly hydrophobic TM domain with positive charges on the C-terminal side. PFIT_bin00500 and PFIT_0835500 are no exception, and this arrangement would result in the highly conserved C-terminal end of the RIFINs being intracellular and protected from antibody recognition.

The highest PfEMP1 expression for IT4CD36ICAM1 is PFIT_0811500. While being on the same chromosome (Chr8) as the dominantly expressed RIFIN (PFIT_0835500), these sequences are about a million bases apart. Based on the genome of the IT4 parasite, the downstream RIFIN gene to PFIT_0811500 is PFIT_0811700 (annotated as a RIFIN pseudogene) while the PfEMP1 upstream of PFIT_0835500 is PFIT_0811700. This may suggest that the RIFIN most actively transcribed may not necessarily be “paired” with the highest transcribed PfEMP1. As the other dominant *rifs* identified to date (PFIT_bin05750 and PFIT_bin00500) have not been mapped to a particular chromosome, it is difficult to speculate further on this possible co-expression of neighbouring *rif* and *var* genes but future identification studies will shed more light on this subject.

The finding that RIFINs may be highly transcribed and expressed in cytoadhesive (but non-rosetting) parasite lines suggests that the role of this highly diverse parasite adhesins are not restricted solely to blood group antigen binding. Future studies alluding to their involvement in other aspects of malaria pathology could yield further insights into the role of malaria in shaping population genetics beyond that of the ABO blood groupings. This study demonstrates the specificity of three polyclonal anti-RIFIN IgG antibodies and reiterates the importance of choosing the right antibodies for the right assays. Given the diversity of RIFIN polymorphisms and the challenges in assaying for them, it is of particular importance that multiple well-validated reagents/techniques be employed to ascertain the presence and involvement of RIFINs in malaria pathology.

## Materials and Methods

### Ethics statement

Experiments were performed in accordance with relevant guidelines and regulations. Serum and RBCs used for parasite culture were collected from Karolinska Hospital blood bank (ethical permit number: 2009/668-31/3) as approved by the Regional Ethical Review Board in Stockholm, Sweden.

### Parasite culture

Rosetting *P. falciparum* strains of FCR3S1.2 (S1.2R)^34^, Palo Alto _varO_ (PAvarO)^35^ and R29^36^, as well as cultures of non-rosetting FCR3S1.2 (S1.2NR)^1^, NF54CSA^37^, FCR3CSA^37^, 3D7CD36ICAM1^38^ and IT4CD36ICAM1^39^ were cultured continuously in blood group O+ erythrocytes, grown in media supplemented with 10% A+ sera and synchronized weekly as described elsewhere^1^,^40^. To minimize the effects of antigenic switching, parasites were cultured for no more than a month after thawing/panning. Cell lines routinely tested negative for mycoplasma contamination.

### SDS extraction of RIFINs and SDS-PAGE

Cultures of late-stage parasites (35–45 hpi) were first treated with 0.05% saponin and washed twice with PBS to enrich for parasites. The pellet was then resuspended in 1 ml of 0.5% Triton X and spun down hard. The supernatant was discarded and the pellet was resuspended in 2% SDS before being spun down hard again. The SDS extract was then used for SDS-PAGE while the pellet was discarded. The SDS extracts were mixed with 4× NuPAGE LDS loading buffer (Invitrogen) and NuPAGE sample reducing agent (Invitrogen) in 1:15 v/v ratio, heated at 100 °C for 10 min, cooled and loaded into the wells of the pre-cast NuPAGE Novex 4–12% Bid-Tris gels (Invitrogen). NuPAGE MOPS running buffer (Invitrogen) was used, as well as pre-stain plus (Fermentas) protein ladder.

### Antibody production

The various antigens used in the animal immunizations are listed in Supplementary Table S1 and immunizations were carried out commercially (Agrisera, Vännäs, Sweden) with Freund’s incomplete adjuvant. Peptide antigens were coupled to KLH prior to immunization. Serum was harvested after the fourth immunization and IgG purified as described before^1^.

### Western Blot

Wet-transfer was performed in Tris-glycine transfer buffer (25 mM Tris, 192 mM glycine, 20% MeOH and 0.025% SDS) on a nitrocellulose membrane. Membrane was blocked overnight in 1% Western Blocking Reagent (Roche) in TBS. Labelling was performed at 10 μg/ml of primary antibody with the exception of anti-PfHsp70 (BioSite SPC-186C/D) that was incubated at a 1:2000 dilution. After 1 hr incubation at RT, membranes were washed three times in TBST and incubated with the corresponding HRP-conjugated ECL anti-rabbit IgG (GE Healthcare) or anti-goat IgG (Life Technologies) antibodies (both 1:5000 dilution) for a further 1 hr. After three additional washes with TBST, Amersham ECL prime western blotting detection reagent (GE Healthcare) was added and blots developed on Amersham Hyperfilm ECL (GE healthcare).

### Air dried monolayers preparation, immunofluorescence staining and microscopy

15-well (4 mm) glass slides (Thermo Scientific) were pre-treated with poly-L-lysine (0.1 mg/ml, Sigma) for five minutes before being left to dry. Parasite cultures were diluted to 0.1% hematocrit in culture media and 10 μl was added to each well for 30 min. The excess media was removed and the cells left to air dry overnight at RT. Samples were then blocked with 2% BSA for 30 min and primary antibodies (8–10 μg/ml) were added for 1 hr at RT. After washing 3× with PBS, the relevant Alexa488-conjugated secondary antibodies were added for a further 1 hr. After another three washes with PBS, 2 μl of Vectashield with DAPI (Vector laboratories) was added to each well and nail polish was used to seal the coverslip. Cells were visualized with Nikon Eclipse 80i fluorescence microscope under oil-immersion at 100× magnification.

### Peptide array

Custom ultra-dense peptide microarrays obtained in collaboration with Roche-Nimblegen were applied for epitope region mapping as described before^41^,^42^. An array containing 175,000 peptides of 12 amino acids in length and with an 11-residue overlap was designed to cover the PfMC2TM family (3D7 and IT background), PHISTs (3D7), RIFINs (3D7 and IT, without pseudogenes), STEVORs (3D7 and IT, without pseudogenes or STEVOR-like proteins), SURFINs (3D7 and IT) and several PfEMP1s (3D7var2csa, ITvar60, ITvar09, PAvar0, TM284var1, HB3var6, 3D7var4, FCR3S1.6) (Supplementary Data S3). Duplicated (conserved) protein sequences were deleted to reduce the total number of peptides. IgG from non-immune rabbit and goat, as well as IgG from rabbits and a goat immunized with various RIFIN peptides/proteins (Supplementary Table S1), were added to individual peptide arrays. Binding analysis of anti-RIFIN antibodies and also control antibodies occurred via secondary Alexa Fluor 647-conjugated anti-goat IgG or DyLight649-conjugated anti-rabbit IgG (both Jackson Immunoresearch) and slide scanning at 2 μm resolution (MS200, Roche NimbleGen Inc., Madison, WI). Each spot on the array was subjected to pre-filtration criteria (detailed in a submitted manuscript) to define reactivity and also minimize false positives: by requiring the spot MFI to be above two times the local spot background MFI and maximum 50% coefficient of variation within the spot. Since the peptides have one amino acid lateral shift between adjacent peptides, if at least two adjacent reactive peptides were reactive, the region (epitope) was regarded bona fide (i.e. requiring a maximum minimal epitope of 11 amino acids). Thus, any reactive peptide with no reactivity on either adjacent peptide was excluded to minimize the number of potential false-positives arising from non-specific binding. The total number of peptides belonging to an epitope region was then used to score the likelihood of specific interaction between the serum-derived polyclonal IgG and various parasite proteins; here termed “reactivity score” for convenience.

### RNA extraction

Synchronized parasite cultures were staged by Giemsa thin smears and harvested at 10, 20, 30 and 40 hpi. For every 100 μl of packed cells, 1 ml of TRIzol reagent (Ambion) was used and the aqueous phase was retained after mixing with chloroform and centrifugation. A second phase separation was performed by addition of acid phenol chloroform (1:1 v/v ratio) following the manufacturer’s recommendations. Precipitation of RNA was done using isopropanol and the pellet was eventually dissolved in RNAse-free water and stored at −80 °C.

### RNA sequencing

The extracted RNA was then checked for quality in Bio-analyzer (Agilent) and 2 μg of RNA used to construct sequence library using TruSeq Stranded mRNA Library Prep kit (Illumina). Sequencing was performed on the Illumina Hiseq 2000 platform to obtain 2 × 100 bp paired-end reads. Reads were aligned to the corresponding reference genomes using Star and HTseq. NF54CSA was aligned to the 3D7 genome while S1.2NR, FCR3CSA and IT4CD36ICAM1 were aligned to the IT4 genome obtained from www.plasmoDB.org. Results are presented here in Reads Per Kilobase of transcript per Million mapped reads (RPKM).

### Quantitative Real-Time PCR (qPCR)

Total RNA was extracted at 20 hpi, DNase treated (Ambion) and reverse transcribed (iScript^TM^, Bio-Rad) according to manufacturer’s instructions. Triplicate amplification reactions were performed for each gene with a mix of 1 μl cDNA, 1× SYBR green mix (PowerUp^TM^, Life Technologies) and 500 nM of both forward and reverse primer (Supplementary Table S3). The program used was as follows 95 °C for 3 min, 50× at 95 °C for 10 s, 60 °C for 30 s with the data recorded at the elongation step. Data was analyzed by computing efficiency corrected relative quantities using the *fructose-bisphosphate aldolase* (FBA, PFIT_1446000) as reference gene and the S1.2NR parasite used as a calibrator (software used: Bio-Rad CFX Manager 2.1). qPCR based relative quantities (RQ) for all time points and genes were thereafter plotted against fold-changes obtained by RNA sequencing and a correlative index was computed by Pearson’s R.

## Additional Information

**How to cite this article**: Ch’ng, J.-H. *et al*. Epitopes of anti-RIFIN antibodies and characterization of *rif*-expressing *Plasmodium falciparum* parasites by RNA sequencing. *Sci. Rep.* **7**, 43190; doi: 10.1038/srep43190 (2017).

**Publisher's note:** Springer Nature remains neutral with regard to jurisdictional claims in published maps and institutional affiliations.

## Supplementary Material

Supplementary Figures and Legends

Supplementary Data S1

Supplementary Data S2

Supplementary Data S3

Supplementary Table S1

Supplementary Table S2

Supplementary Table S3
