# Supplementary material for: Epitopes of anti-RIFIN antibodies and characterization of rif-expressing Plasmodium falciparum parasites by RNA sequencing
Source: Sci Rep. 2017 Feb 24;7:43190. doi: 10.1038/srep43190 (PMC5324397; doi:10.1038/srep43190)

Epitopes of anti-RIFIN antibodies and characterization of *rif*-expressing *Plasmodium falciparum* parasites by RNA sequencing

Jun-Hong Ch'ng, Madle Sirel, Arash Zandian, Maria del Pilar Quintana, Sherwin Chun Leung Chan, Kirsten Moll, Asa Tellgren-Roth, IngMarie Nilsson, Peter Nilsson, Ulrika Qundos & Mats Wahlgren

Supplementary Figure Legends

Supplementary Figure S1. Full length western blots of RIFINs in multiple parasite strains. SDS-extracted parasite lysates of (A) S1.2R, S1.2NR, FCR3CSA, NF54CSA, 3D7CD36ICAM1 and IT4CD36ICAM1 were run on SDS-PAGE (lanes 1-6 respectively), as well as SDS-extracted lysates of (B) PAvarO and R29 (lanes 1 and 2 respectively). Blots were transferred to nitrocellulose membrane and blotted with rabbit anti-PfHsp70 (1:2000), non-immune rabbit IgG (10 µg/ml), $R\alpha RIF_C$ rabbit IgG (10 µg/ml), $R\alpha RIF_I$ rabbit IgG (10 µg/ml), non-immune goat IgG (10 µg/ml) and GαRIF goat IgG (10 µg/ml). Corresponding HRP-conjugated secondary anti-rabbit and anti-goat IgG antibodies were used together with ECL reagent for detection. Description of the antigens used for immunizations of $R\alpha RIF_C$, $R\alpha RIF_I$ and GαRIF are described in Supplementary Table S1.

Supplementary Figure S2. Epitope region mapping of anti-RIFIN antibodies with epitope regions highlighted. The reactivity for IgG purified from (A) $R\alpha RIF_C$, (B) $R\alpha RIF_I$ and (C) GαRIF are shown here against peptides from the RIFIN protein PF3D7_0100400 (left panels) and the corresponding epitope regions (right panels, black bars). Y-axis shows mean median fluorescence intensity of duplicate spots and X-axis shows the peptide number from the N terminus to the C-terminus. $R\alpha RIF_C$ was immunized with the C-terminus, $R\alpha RIF_I$ with the indel region and GαRIF with the full length PF3D7_0100400 protein.

Supplementary Figure S3. Alignment of RIFIN amino acid sequences. Full length sequences of PF3D7_0100400, PFIT_bin05750 (dominant *rif* of S1.2R), PFIT_0835500 (dominant *rif* of IT4CD36ICAM1) and PFIT_bin00500 (dominant *rif* of FCR3CSA) were aligned by Clustal Omega and the three predicted transmembrane (TM) regions highlighted in pink. Amino acid sequences of PF3D7_0100400 and PFIT_bin05750 were used previously in Goel et al. (2015).

Supplementary Figure S1

A.

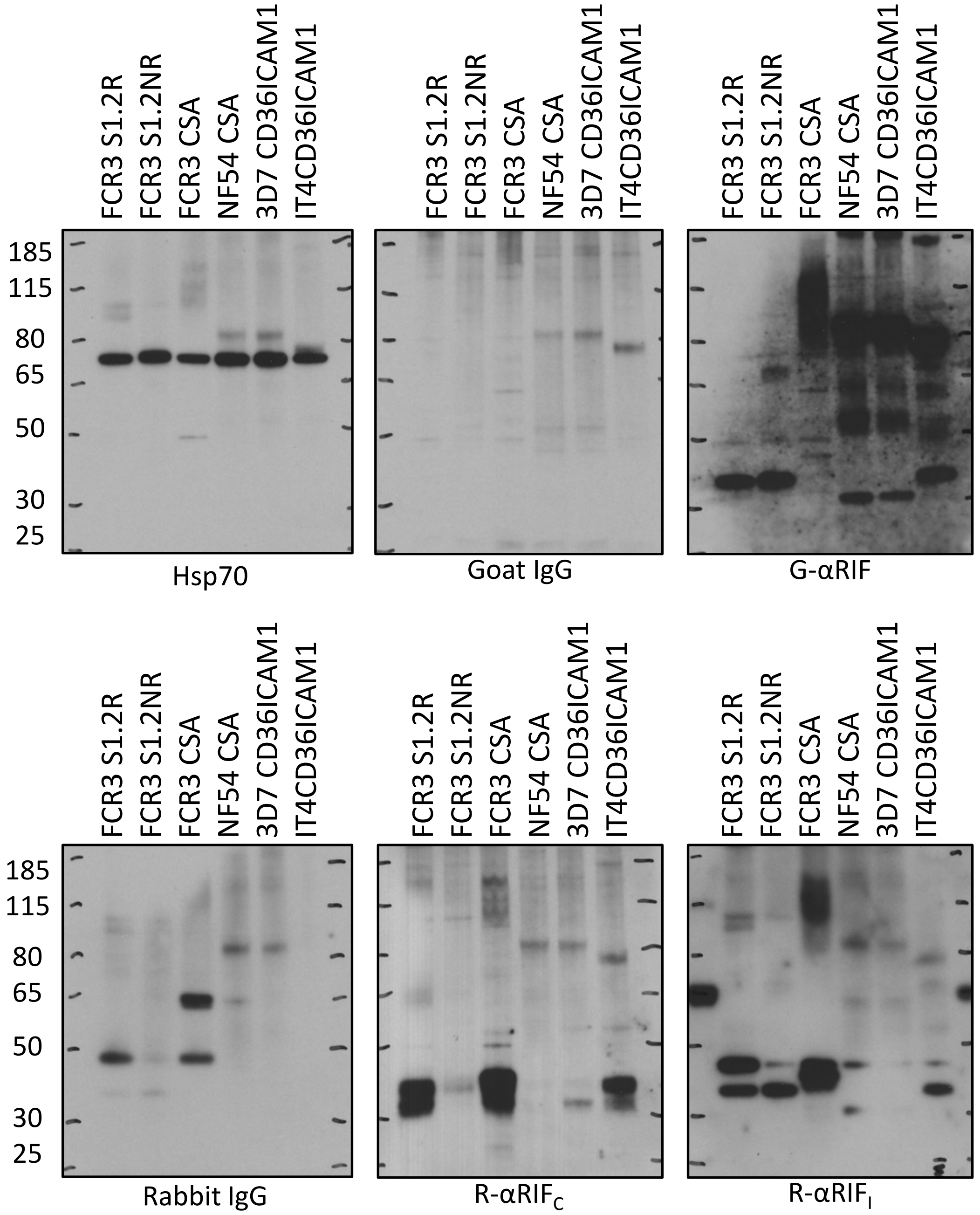

B.

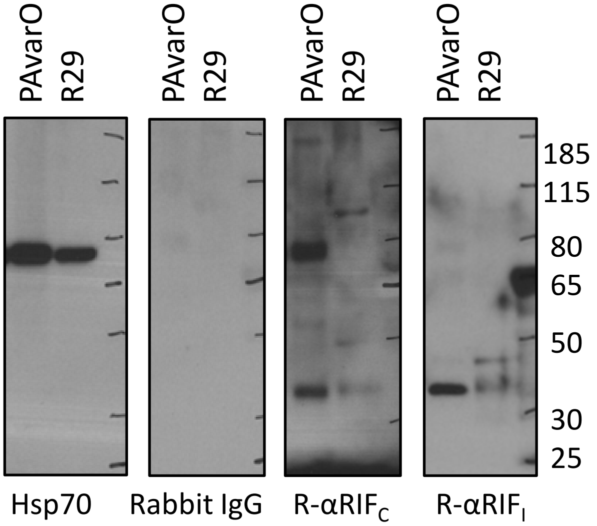

Supplementary Figure S2

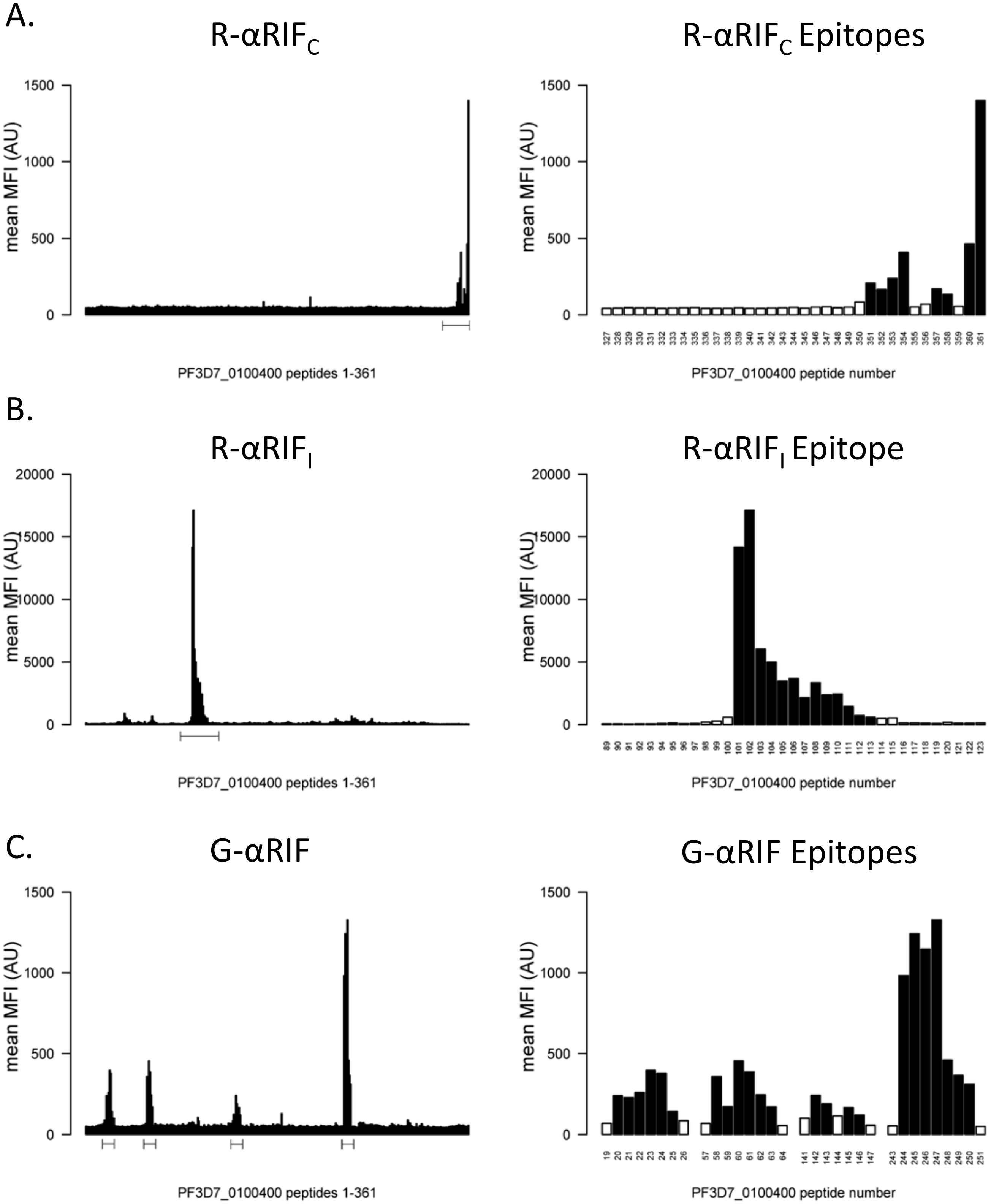

Supplementary Figure S3

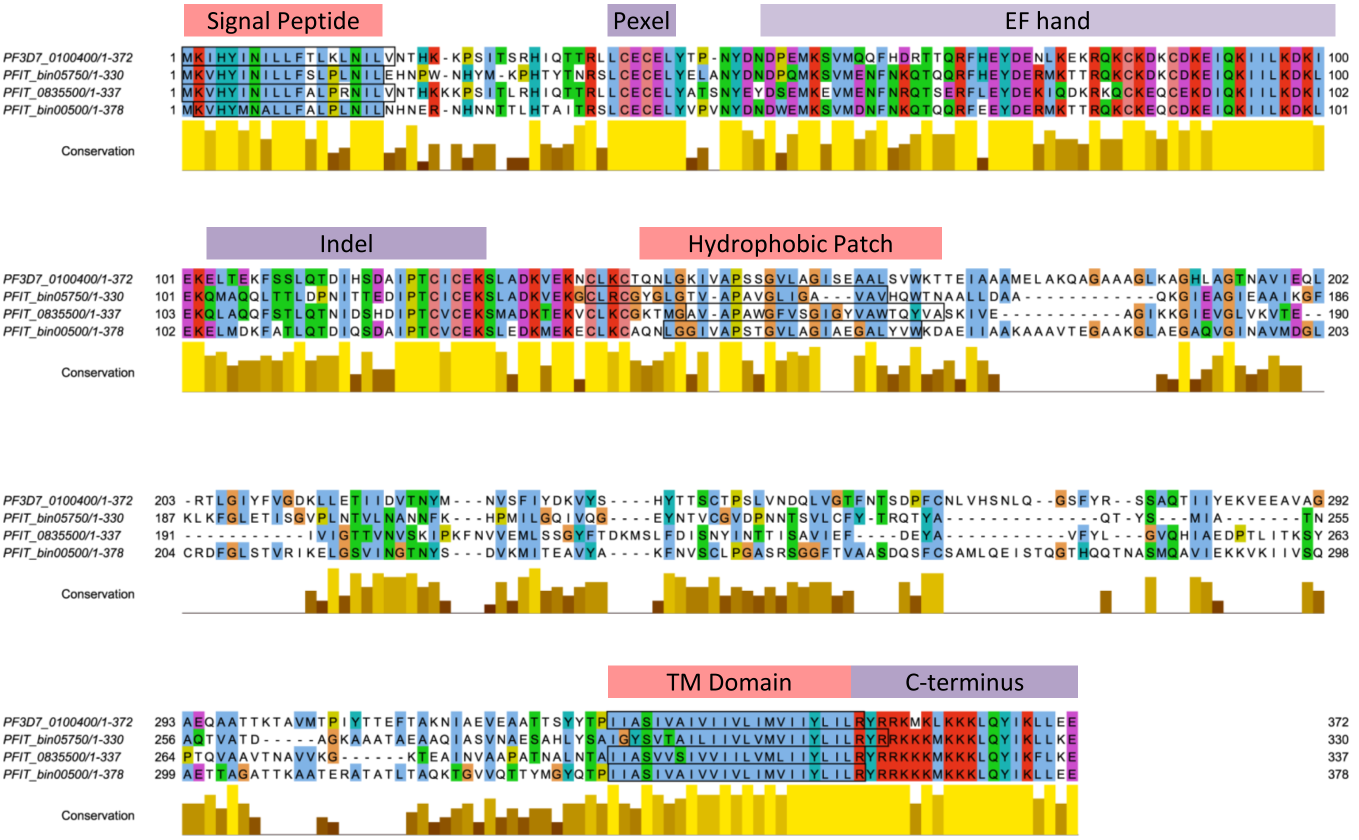

Supplement: Supplementary Figures and Legends [file srep43190-s1.pdf]
